# Graph Algorithms for Mixture Interpretation

**DOI:** 10.3390/genes12020185

**Published:** 2021-01-27

**Authors:** Benjamin Crysup, August E. Woerner, Jonathan L. King, Bruce Budowle

**Affiliations:** 1 Center for Human Identification, University of North Texas Health Science Center, 3500 Camp Bowie Blvd., Fort Worth, TX 76107, USA; august.woerner@unthsc.edu (A.E.W.); jonathan.king@unthsc.edu (J.L.K.); Bruce.Budowle@unthsc.edu (B.B.); 2 Department of Microbiology, Immunology, and Genetics, University of North Texas Health Science Center, 3500 Camp Bowie Blvd., Fort Worth, TX 76107, USA

**Keywords:** probabilistic genotyping, mixture interpretation, massively parallel sequencing, mitochondrial mixtures, graph algorithm

## Abstract

The scale of genetic methods are presently being expanded: forensic genetic assays previously were limited to tens of loci, but now technologies allow for a transition to forensic genomic approaches that assess thousands to millions of loci. However, there are subtle distinctions between genetic assays and their genomic counterparts (especially in the context of forensics). For instance, forensic genetic approaches tend to describe a locus as a haplotype, be it a microhaplotype or a short tandem repeat with its accompanying flanking information. In contrast, genomic assays tend to provide not haplotypes but sequence variants or differences, variants which in turn describe how the alleles apparently differ from the reference sequence. By the given construction, mitochondrial genetic assays can be thought of as genomic as they often describe genetic differences in a similar way. The mitochondrial genetics literature makes clear that sequence differences, unlike the haplotypes they encode, are not comparable to each other. Different alignment algorithms and different variant calling conventions may cause the same haplotype to be encoded in multiple ways. This ambiguity can affect evidence and reference profile comparisons as well as how “match” statistics are computed. In this study, a graph algorithm is described (and implemented in the MMDIT (Mitochondrial Mixture Database and Interpretation Tool) R package) that permits the assessment of forensic match statistics on mitochondrial DNA mixtures in a way that is invariant to both the variant calling conventions followed and the alignment parameters considered. The algorithm described, given a few modest constraints, can be used to compute the “random man not excluded” statistic or the likelihood ratio. The performance of the approach is assessed in in silico mitochondrial DNA mixtures.

## 1. Introduction

The guidelines and interpretation strategies of mixed DNA profiles (multiple individuals in one sample, such as a stain comprised of blood from two people) is one of the most pressing open questions in modern forensic genetics. Continuous methods for mixture interpretation require quantitative information (such as signals represented as peak heights with capillary electrophoresis analyses or read count with massively parallel sequencing), while semi-continuous and binary methods neglect such data (for review [1]). The forensic genetic literature is largely focused on autosomal short tandem repeats (STR, but see [2,3,4,5]) and other cases where a locus is assessed as a single unit of analysis (DNA fragment, MPS (Massively parallel sequencing) read) that is determined holistically (as a haplotype). While interpreting autosomal STRs has inherent complexity (e.g., negative and positive stutter), there are additional and different considerations to interpreting mitochondrial DNA mixtures. Some of the complexities stem from nuclear mitochondrial insertions (Numts, [6,7]), which may be polymorphic in populations [8], and can present as what could be interpreted as minor alleles that confound mixture interpretation (but see [9,10,11,12]). Another issue is heteroplasmy (where a single individual possesses multiple mitochondrial genomes with different haplotypes), which can also present as a type of DNA mixture and (of practical importance) can vary substantially between and within body sites [13,14] and even within control samples [15,16]. The implications are that a reference from some individual may not exactly match an item of evidence even if the two samples originate from the same person, which is in part argued by run-to-run variant frequency variability [15]. However, even in the absence of the complexities of Numts and heteroplasmy, there are further considerations for mitochondrial DNA mixture interpretation. One major issue is that a mitochondrial DNA haplotype may be comprised of potentially 100 s of amplicons. These amplicons may overlap to varying degrees and may have varied PCR efficiencies [17]. In contrast, continuous methods based on massively parallel sequencing (MPS) data need to balance purely quantitative data (variant read-counts) against the linkage (and resultant linkage disequilibrium, LD) inherent to the mitochondrial genome (e.g., as per [18]). Thus, there is the potential for contention between the phylogeny (which may favor linkage of the major and minor allele at a pair of sites) and the quantity of reads that support some allele call set (which may favor the opposite). The preferred interpretation depends on several factors: allele- or amplicon-specific bias in the PCR efficiency would suggest favoring the linkage interpretation, while homoplasy would suggest the opposite.

An additional concern is ensuring that the variant calls are phylogenetically consistent (as per [19,20]). Phylogenetic alignment and variant calling will, in the vast majority of cases, give variant call sets that are stable in time. There are exceptions, however, and the exact standards of what is phylogenetically optimal are evolving (e.g., [21]). Any change in the variant calling procedures (with respect to the database samples) creates a potential for error and bias unless the appropriate algorithms are employed. Additionally, full descriptions of how phylogenetic variant calling is to be applied to mixed profiles are ongoing, further complicating the application to mixed samples.

Many of these issues can be side-stepped if the signal intensities are simply ignored. Such approaches permit the direct assessment of DNA mixtures without quantitation [3,22]: while doing so may reduce the overall power of the approach, the resulting methods involve fewer complicating factors. However, the typical means of describing mitochondrial DNA variation differs from how STR and haploid mixtures are evaluated (e.g., [3,4,23]). STRs are often described either as a nucleotide sequence or as the length of that sequence. These descriptions are invariant to the reference genome and directly describe 10 s or 100 s of nucleotides. In contrast, genomic variation (mitochondrial or otherwise) describes sequence differences with respect to a reference at the level of either a single nucleotide (single nucleotide variants, SNV) or a small range of nucleotides (multiple nucleotide variants, MNVs). This difference in description stems from the chemistry in which typical read lengths are too short to span the mitochondrial DNA haplotype. These two descriptions are not equivalent. One major distinction is that a single haploid allele call can be associated with multiple sequence differences. Consequently, two sets of sequence differences can neither be used to say that a reference profile matches some individual [19], nor can the probability of this match be assessed in some database (as per SWGDAM (The Scientific Working Group on DNA Analysis Methods) recommendations [24]). Changes to the methods for alignment and variant calling are a major reason for this phenomenon: considering that allele calling conventions for mitochondrial DNA mixtures have yet to be well developed, this point is especially pertinent.

For single-source mitochondrial DNA match statistics, the recommended approach does not directly compare the variant calls, but instead composes strings from those variant calls and performs searches and comparisons on those strings in a manner that is alignment free [19,21]. This approach is well-founded; as long as the haploid variant calls are described without error, the underlying haplotypes will be deemed to match if (and only if) the haplotypes are identical. String-to-string comparison thus serves as the main engine of the match statistic computation, both when comparing evidence to a reference and when searching through a database. However, this approach only applies to single-source data. No method to-date has been described that can perform the analogous computation on mixtures.

In this paper, graph algorithms are introduced to assess if a haplotype is consistent with some set of variants in order to compute the “random man not excluded” (RMNE) statistic [5]. In addition, the question of whether or not a set of haplotypes can exactly explain some set of variant calls can be addressed, which in turn permits the computation of likelihood ratio (LR) statistics. The algorithm described is binary in that it uses allele calls which are assumed to be measured without error while neglecting the quantitation of the calls. Of note, both the LR and the RMNE approaches are invariant to how the variation itself is encoded (given some constraints), and they apply to insertion and deletion polymorphisms regardless of the alignment strategy.

## 2. Materials and Methods

### 2.1. Directed Acyclic Graphs and Sequence Variation

Sequence alignment and variant calling is parametric. When applied to mitochondrial DNA variation, different alignment parameters and variant calling conventions may characterize the same haplotype in multiple (and different) ways. Mitochondrial DNA variant calling is perhaps more challenging than characterizing autosomal variation as the rules of phylogenetic alignment and variant calling need not involve the optimal pairwise alignment to the reference sequence, but instead consider the alignment in a phylogenetic context [19,21]. The SNV calling rulesets, though generally stable, may be subject to change over time. Further, they may not necessarily be strictly followed, especially if all that is required is the description of the underlying haplotypes. As different variant call sets may be used to describe the same underlying haplotype, the forensic interpretation of single-source mitochondrial DNA profiles should be performed using string-to-string comparisons to ensure correctness [19]. Herein, the analogous approach is described for mixtures. In particular, a mixture can be represented computationally as a graph, and “match” statistics (herein, the RMNE or the likelihood) on this graph can be computed by comparing haplotype strings to this graph. To provide some background, in discrete mathematics a graph is defined as a collection of vertices (*V*, also called nodes or points) and a collection of edges (*E ε V* × *V*, or links). Elements in *E* in turn relate pairs of nodes in *V*, connecting some vertexes but perhaps not others. *E* can be directed, e.g., permitting v_i_ to connect to v_j_ but not necessarily vice versa and can describe cycles, e.g., connecting v_i_ to v_j_ to v_k_ and back to v_i_, or *E* can be constrained to disallow cycles. One common type of graph is a DAG (Directed acyclic graph), or a directed (edges denote direction) acyclic (no cycles are allowed) graph (collection of vertices and edges).

**Figure 1:**
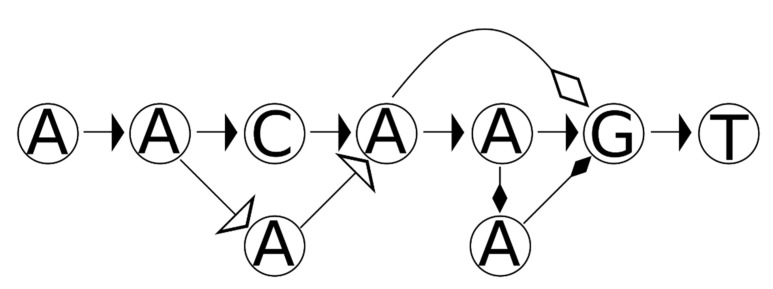
A DNA sequence and sequence variants represented as a directed acyclic graph (DAG). A DNA sequence (AACAAGT) can be thought of as a collection of single nucleotide vertices connected by directed edges (arrows). If AACAAGT is considered as the reference sequence (horizontal path), sequence variants (differing arrows) can be thought of as directed edges that depart from the horizontal path. The A deletion (open diamond arrow) is depicted, as well as a C to A transversion (open arrow), and as well as an A insertion between the A and the G (solid diamond arrows).

DNA sequences can be represented as DAGs (Figure 1). Taking a reference sequence as an example, consecutive letters in the reference can be thought of has having an implicit directed edge between nodes (nucleotides) (Figure 1). Sequence variation can also be encoded in DAGs, which can easily represent mismatches, insertions and deletions. Indeed, one of the trends in modern approaches to read mapping is to consider both the reference sequence and reference variation by mapping (aligning) reads from MPS to DAGs (when structural variants are not considered, e.g., [25,26]), while other approaches permit cycles, e.g., [27,28] (see also [29], which may apply well to STRs). In such approaches, a read is aligned to any path (consecutive nodes connected by directed edges) in the graph. Taken from a different perspective, the DAG can be thought of as a sequence or haplotype generator, wherein any path that begins at the first character and finishes at the last character (which, in practice, can be leading and sentinel values, e.g., the empty string) can be thought of as a feasible DNA sequence that matches the graph.

### 2.2. Mitochondrial Mixtures as Variation Graphs

An appreciated difficulty in forensic mitochondrial genomics is the assessment and treatment of forensic match statistics. Match statistics in this context are taken to mean those statistics that apply to mixtures, and in particular, the RMNE and its probability, as well as the likelihood as applied to the likelihood ratio. While STRs are described by allele calls (either as numbers or character strings), mitochondrial DNA haplotypes are often described as sequence differences (typically, with respect to the revised Cambridge reference sequence (rCRS [30]), which graphically correspond to the non-primary edges (horizontal path) in Figure 1. As a reminder, sequence differences describe the operations necessary to transform one string (the reference sequence) into another (the mitochondrial DNA haplotype of some individual or sample). However, sequence differences are not necessarily directly comparable [19,21]; two sequences may appear different yet may describe the same mitochondrial DNA haplotype. As an example, the sequence AACAGT can be encoded as a deletion of either the 4th or the 5th A in the reference sequence AACAAGT (as per Figure 1), thus encodings of 4 and 5 del are equivalent (i.e., when applied to the reference they yield the same strings), though they are not equal (when evaluated as sets). One strategy to assess if two mitochondrial DNA haplotypes match is to decompose both sets of sequence variants into strings and then to perform match statistics on the resulting nucleotide sequences. This approach is of particular importance to assess equality and near-matches (as per [19]), as may be important for determining inconclusive calls that may indicate genotyping errors or near maternal relatives (though “near” may in fact be quite distant [31]).

One way in which mitochondrial DNA mixtures are represented is the same encoding style as that of single-source samples. That is, the mixture is presented as a set of sequence differences with respect to the reference. This encoding, however, appears to contain some of the same problems as were apparent in single-source descriptions. For example, consider a two-person mixture that is genotyped without error, and two single-source samples (e.g., a victim and a suspect) are likewise genotyped, can it be said that the two single-source samples match (or comprise) the mixture? By logic already provided, the two sets of sequence differences (describing the mixture, set 1, vs the two single-source samples, set 2) are not directly comparable (i.e., they may differ and still describe the same strings). Further, to assess the hypothesis probabilistically, a database of single-source haplotypes should be considered to establish the rarity of some match (e.g., using an approach derived from [3]).

To better understand this problem, consider a two-person mixture. For simplicity consider just three sites encoded in the fashion of EMPOP [32]: 73G 154W 178M (W = AT, M = AC) and neglect the possibility of indels (insertion or deletion polymorphisms). Further, assume that the sites are genotyped without error, and there is no (measurable) heteroplasmy. From this encoding it appears that both individuals differ from the reference sequence at position 73 (both individuals are G), while the IUPAC (International Union of Pure and Applied Chemistry) ambiguity codes [33] suggest that the two individuals differ between each other at positions 154 and 178. Under the given conditions, there are two possible (unordered) single-source haplotypes that would explain this mixture: the haplotype pair (73G, 154A, 178A; 73G, 154T 178C) or the haplotype pair (73G, 154T, 178A; 73G, 154A 178C). Thus, one approach to say that two single-source mitochondrial DNA haplotypes can explain some mixture would be to consider both pairs, construct the DNA sequences (apply the difference operations), and call a “match” if, for example, the victim and the suspect match both sequences (of either the first or the second pair).

While the above algorithm would preserve correctness—mitochondrial DNA sequences, not their difference encodings, are compared, and matches consider either configuration (the first pair or the second pair) that is feasible from the mixture—the algorithm described scales exponentially with the number of variants and thus would fail in mitochondrial genomics applications. As an example, if an additional IUPAC code were to be considered, the number of feasible haplotype pairs (2) would double (4), as each pair would need to consider both orientations of the new ambiguous site. In practice, the LD inherent to the mitochondrial genome will reduce the number of possibilities (e.g., excepting private mutations, in the absence of recombination and recurrent mutation there is only 1 possibility); however, how this LD can be appropriately captured is an open question. More generally, if two-person mixtures are considered, the number of feasible haplotype-pairs is 2^Number of ambiguous sites-1^, which for a mixture with 31 ambiguous sites would result in more than a billion possibilities. Given that the mean number of pairwise differences between mitochondrial genomes in Caucasians is ~30 and in African Americans is ~55 [34], the approach outlined of enumerating all possible haplotypes from a variant graph is effectively infeasible.

An alternative approach is to construct a variation graph which describes the mixture. One strategy for doing so is to treat the reference sequence as a template and then apply each difference operation (substitute, insert, or delete) to the graph in turn. Applying this logic to mismatches is simple. Considering again the 73G 154W 178M example, 73G would change the 73rd nucleotide position on the rCRS to G and would add edges (as per the non-horizontal arrows in Figure 1) for the two ambiguity codes. The operations are very nearly described in the style of EMPOP (see Appendix A). Applying the appropriate logic to insertion or deletion (indel) polymorphisms requires, however, more nuance.

### 2.3. Equivalences between Sequences and Variant Graphs

The manner in which the graph is constructed requires some additional inspection. First, consideration must be given so that, if (for a given base or sequence) the reference sequence is not present in the mixture, it is also not present in the graph. Second (and more involved and of particular interest to this work), if the graph is built in a specific manner, a simple test can be devised to determine if a given set of individuals is consistent with (i.e., matches) the mixture.

**Figure 2:**
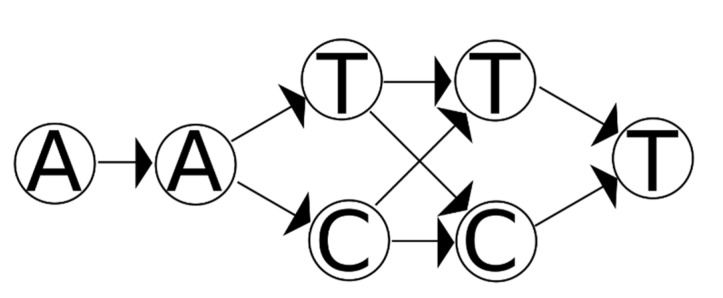
A variant graph for a mixture with two different bases at two adjacent sites. There are four possible sequences that can match this graph (AATTT, AATCT, AACTT, AACCT). All of the nodes in this graph describe sequence data that were in the mixture. Consequently, a suspected set of contributors must (collectively) represent every node in this graph.

A necessary condition for whether a set of haplotypes is consistent with the mixture is for each haplotype to be represented by a path through the graph (possibly allowing for a small number of errors if fuzzy matching is sought). Additionally, if the haplotypes created the mixture described by the graph, then the paths taken by the sequences of the haplotypes should collectively cross every node in the graph: put another way, all of the bases in the graph should be accounted for by the haplotypes. As an example, the sequences AATCT and AACCT are both consistent with the variant graph in Figure 2, but the two sequences together could not have produced the mixture represented by this graph (the individuals only have a C in the fourth base, but one of the contributors to the mixture has a T) excluding genotyping error.

**Figure 3:**
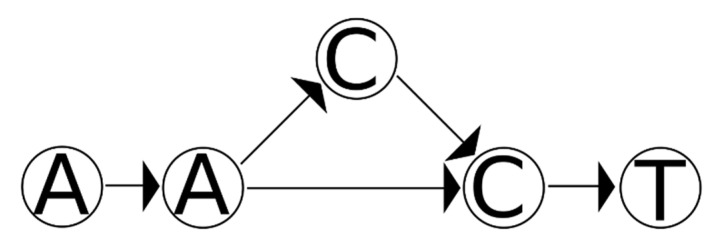
A variant graph for a mixture of two individuals involving an indel. A person of interest (POI) (AACCT) is mixed with another POI (AACT), and a variant graph is created to describe this mixture. A matching set of contributors must account for every node on this graph. Additionally, all edges must be accounted for if the indel is considered correctly.

However, these two conditions on their own are not sufficient to correctly handle all cases. As an example, Figure 3 shows a graph describing a mixture of two haplotypes, one with the reference sequence and one with an insertion. If the two haplotypes in question both have the insertion, then both haplotypes are consistent with the graph and their sequences collectively hit every node in that graph. However, they could not produce the mixture represented by the graph since neither of them has the reference sequence.

An obvious additional constraint is to require that all edges are collectively traversed by the sequences. However, this constraint does not quite work. As an example, suppose there are two SNVs adjacent to each other (see Figure 2). If the variant data have no phase information (and phylogenetic priors are ignored), each node for the first variant must link to both nodes for the second variant; using an edge constraint would imply at least a four-person mixture.

The main reason for this problem is that there is uncertainty in the edges of the graph. It is certain (from an algorithmic perspective) that every node in the graph represents sequence that was present in the mixture. However, it is uncertain whether some of the edges are present in the mixture: TT, TC, CT, and CC are all possible edges. However, without locally deconvolving the variants it is unknown which sets were actually present. It would be useful in some way to express this uncertainty without adding edges that may or may not actually be present.

**Figure 4:**
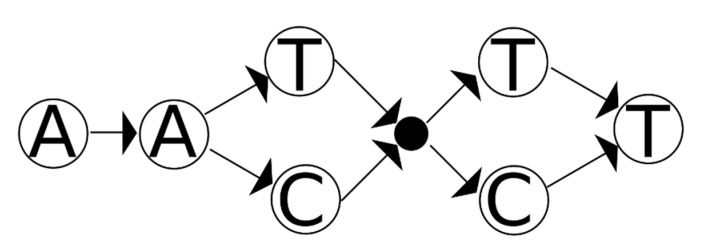
A remade version of the graph in Figure 2, using an epsilon node. In this version of the graph, all edges are known to be in the mixture. Note that this graph can be turned into the one from Figure 2 by examining all nodes reachable from a given node without consuming sequence and adding (uncertain) edges.

A way to overcome this issue is to allow for nodes that do not correspond to a base (referred to here as “epsilon” nodes). These nodes, when used in a path through the graph, do not use any of the sequence characters and can be used to bottleneck the paths through the graph. Instead of having links between two separate tracks, the two tracks can converge on an epsilon node which then branches out. As an example, the graph from Figure 2 can be recast as the graph in Figure 4.

If all nodes and edges in a graph are known to be present in the mixture, then a test for whether a set of haplotypes is consistent with the mixture is to determine whether the haplotypes collectively account for every node and edge in the graph (along with a requirement that the sequences are individually consistent with the graph).

Whether or not there are uncertain edges (or nodes) depends heavily on the manner used to construct the graph. There are cases that obviously have uncertain edges: for instance, when there is more than one path through a set of epsilon nodes between the same starting and ending nodes. However, there is no simple test for uncertain paths; in certain situations (starting with more informative input data) the graph in Figure 2 might be more accurate than the one in Figure 4. The correct graph depends on the input data (and the respective interpretation).

**Figure 5:**
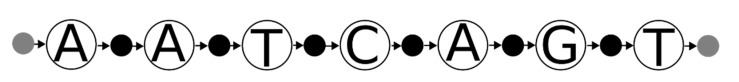
A reference sequence with epsilon nodes (black circles) added between each base (as well as terminal epsilon nodes, in gray, added to denote the start and end).

The convention used in this work starts with the reference sequence and adds an epsilon node between each base (see Figure 5).

**Figure 6:**
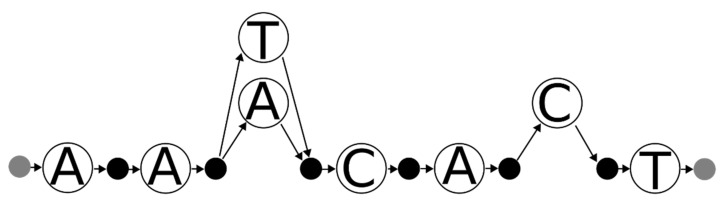
The graph from Figure 5 with three alleles added to it (3T, 3A, and 6C). Note that one of the bases (3T) is the reference sequence. Any variation at a base will delete the reference base.

Any index (or set of indexes) at which a variation is called has the reference node removed and the observed base(s) added in its place (see Figure 6).

**Figure 7:**
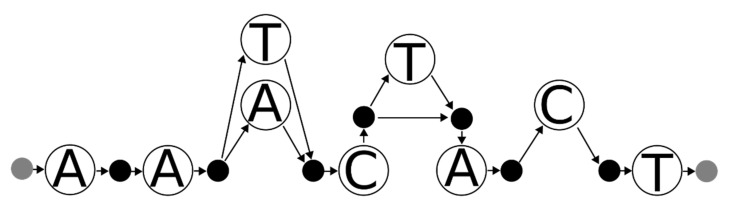
The graph from Figure 6 with an insertion added (4.1T). Note that this insertion adds an additional epsilon node.

Insertion variants will add an epsilon node and the inserted sequence after the epsilon node following the base (see Figure 7).

**Figure 8:**
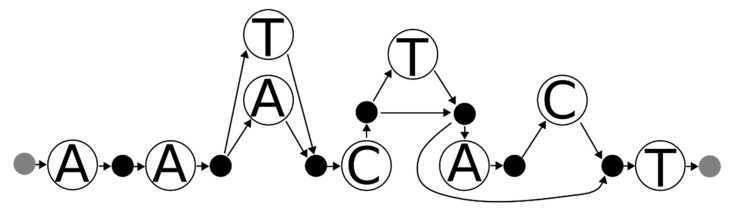
The graph from Figure 7 with two adjacent deletions (5 del, 6 del) added to it. Note that nothing is indicated about the relationship between the insertion and the deletion.

Deletion variants will add a link from the node before the deleted base(s) to the epsilon node after the last deleted base. Adjacent deletions are, for presentation purposes, assumed to be part of the same variant (see Figure 8).

### 2.4. Match Statistics on Graphs

Once the graph has been constructed it can easily accommodate a string (i.e., haplotype) search. Because the variation itself is encoded on the backbone of the reference sequence, variations that are equivalent yet describe the same string also have the same path in the graph (e.g., as per the AACAGT example, it does not matter which A is deleted in the graph; the string that some path describes remains the same). Thus, it is feasible to determine if some individual string sequence (single-source haplotype) is consistent with some mixture, or whether such an individual can be excluded as is conducted with the RMNE statistic.

The addition of epsilon nodes also enables determination of whether or not a set of strings can explain a graph. This approach, in turn, permits the computation of LR statistics (neglecting the theta correction terms), as it can be addressed if, for example, two haplotypes can exactly explain some set of variant calls. Importantly, such an approach also applies to indel polymorphisms. Further, as a necessary condition to a set of sequences matching some graph is that they have a path in the graph. A fast approach to computing LR statistics is to first find those haplotypes in the database that are consistent with the mixture, and of these haplotypes, consider the relevant combinations of them (e.g., pairs, in the case of a two-person mixture with two unknowns) that can explain the mixture. In practice, only a minute fraction of haplotypes should be consistent with the graph, which in turn greatly reduces computation time.

### 2.5. The Random Man Not Excluded

The two commonly accepted approaches contending with DNA mixtures are the LR and the RMNE approach [35]. While both are valid tools for considering and interpreting mixtures, the RMNE, while generally less powerful, has the advantage of applying to arbitrarily complex mixtures without needing to specify the number of contributors in the mixture. More formally, a potential contributor to a mixture can be any individual whose mitochondrial DNA haplotype can trace a path through some variant graph, while the probability of a path (p(RMNE)) can be computed by considering the proportion of individuals that can trace a path in the graph. An upper-bound on this estimated probability of inclusion can be calculated by using method of Clopper and Pearson [36] to correct for a finite database size, with the approach used herein taking a 95% upper-bound on the 2-tailed confidence interval of Clopper and Pearson.

### 2.6. Likelihood Ratios

The LR methodology proposed herein is a simple extension of the LR approach of Ge et al. [3,4] In the approach of Ge et al., a population database is employed, and the number of combinations of haplotypes that can explain the mixture is computed, with the observed haplotypes derived from the database treated as unknown contributors. The likelihood of some set *U* of |*U*| unknown haplotypes (*H*_1_ ... *H*_|*U*|_) in the mixture can be computed naively as:

$$L\left(U\right)=L\left(H_{1},\;H_{2},\;\ldots ,\;H_{\left|U\right|}\right)=\left|U\right|!\overset{\left|U\right|}{\underset{j=1}{\prod }}\mathrm{Pr}\left(H_{j}\right)$$

where Pr(*H_j_*) may be taken as the frequency of haplotype *H_j_*. As there are an arbitrary number of sets *U* that explain the mixture, the final likelihood is that which pertains to the total set *T* of all sets {*U*_1_, …, *U_t_*} that may explain the mixture under some hypothesis:

$$\underset{U_{i}\;\in \;T}{\sum }L\left(U_{i}\right)$$

An alternative approach is to compute theta-corrected likelihoods, which are derived from the sampling formula of Balding and Nichols [37] (as described in [3]) and can be estimated in a similar fashion. A related, and possibly more conservative approach, is to consider (in the case of a two-person mixture) a database of haplotype pairs [4] (herein, termed the GBC likelihood). With the GBC likelihood, the likelihood of some set of mitochondrial DNA sequences is taken as the proportion of pairs (or triples in the case of a three-person mixture) in the database that can explain the mixture, and a Clopper and Pearson upper-bound [36] can serve as an additional correction for the finite size of the database. To motivate the utility of the GBC likelihood, one significant drawback to the classical likelihood estimation approaches is that it is presumed that the database is of sufficient size to be able to explain the mixture in question. Under most sampling strategies, most mitochondrial genomes are unique (as per [34]), thus it follows that there will be no combination of haplotypes in the database that can explain most mixtures. Under these circumstances the classical likelihood (above) is undefined as the set *T* is the empty set, although it could also be said that the likelihood is 0 (as it is an empty sum), with either case being problematic for the purposes. With the GBC estimator, however, when some hypothesized number of *u* unknown contributors is considered and the likelihood when the database cannot explain the mixture is simply an Clopper and Pearson upper bound on $0/\begin{array}{c} n\\ u \end{array}$, where *n* is the database size and $\begin{array}{c} n\\ u \end{array}$ (*n* choose *u*) is the number of ways of choosing *u* haplotypes from the database (i.e., if *u* = 2, then the equation becomes n(n−1)2).

Applying the GBC likelihood approach to variant graphs is straightforward. The variants in the mixture are characterized, and a variant graph composing the mixture is constructed. Any known (single-source) haplotypes are also constructed (e.g., from the victim) and some number of individuals is proposed (for example two). All of the ways of constructing a two-person mixture considering the known haplotypes is then computed (i.e., by adding the two knowns to the database implicitly), and the proportion of times that mixtures can be explained by the haplotypes proposed is calculated. Further optimizations consider not the entire database of unknown haplotypes (which may be large), but just those that cannot be formally excluded (which is often a small number).

### 2.7. Implementation

The algorithms to encode variant graphs as well as perform RMNE and LR statistics are implemented in the open source R package MMDIT (Mitochondrial Mixture Database and Interpretation Tool) (https://github.com/Ahhgust/MMDIT). The core algorithms are written in C++ with the R (>3.6) interface provided by Rcpp [38]. MMDIT is populated by default with 40,644 whole mitochondrial genomes that are derived from the human mitochondrial database (HmtDB [39,40]). Rather than relying on the variant calls from HmtDB, the fasta sequences [41] were re-genotyped with the software Converge (TFS, v.2.1) in an automated fashion (see [11] for details). MMDIT uses an SQLite relational database to store variant calls, as well as the ascribed population labels. It should be noted that the data used herein are from the public domain and may contain some errors, but since the data are used for illustrative purpose the process is not affected.

MMDIT was used to create two-person in silico mixtures. In brief, MMDIT has a database of sequence differences (i.e., variant calls) encoded with respect to the rCRS. These calls are specifically designed for single-source samples and as such describe insertion, deletion and mismatch operations, as well as the position of the variations. In silico mixtures were created from the variant calls, though note that the encoding requirements of the variant graph have subtle differences to that of sequence differences. The largest distinction is that variant graphs require that indel polymorphisms are encoded as single events that describe the alleles present at some genomic location (see Appendix A), an issue that can be problematic to describe in an automated fashion if indels are found to partially overlap or perhaps even abut. As these scenarios are rare, a procedure was designed to randomly sample mitochondrial DNA haplotypes conditioned on there being no partially overlapping indel variation.

### 2.8. Visualizations and Statistics

All visualizations and statistics were computed using the R statistical computing environment [42], and visualizations were created using ggplot2 [43].

## 3. Results and Discussion

**Figure 9:**
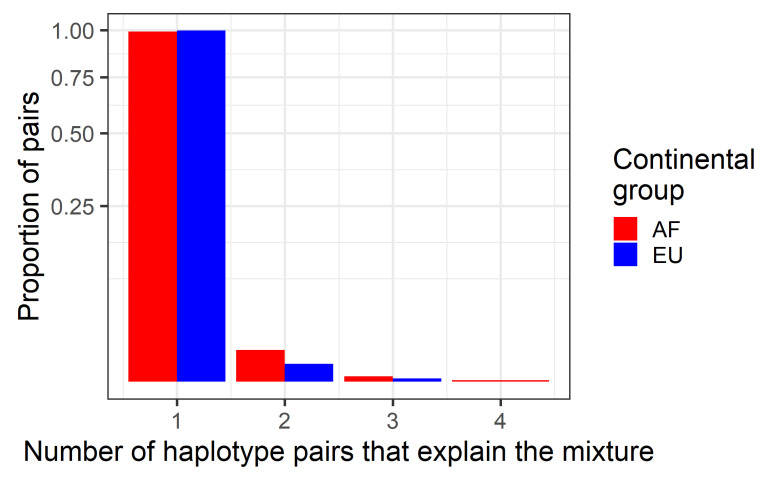
The proportion of haplotype pairs that explain two-person mixtures. Up to 100,000 in silico two-person mixtures were created using haplotypes from the Human Mitochondrial database (HmtDB). Mixtures were created within populations taking pairs of individuals from the African (AF) and European (EU) continental groups (colors). The number of distinct haplotype pairs that can then explain the mixture were tabulated (*x*-axis) as well as the frequency of the occurrence (*y*-axis, square root scale). Haplotype sampling occurred with replacement, thus the minimum number of haplotype pairs that can explain the mixture is one.

**Figure 10:**
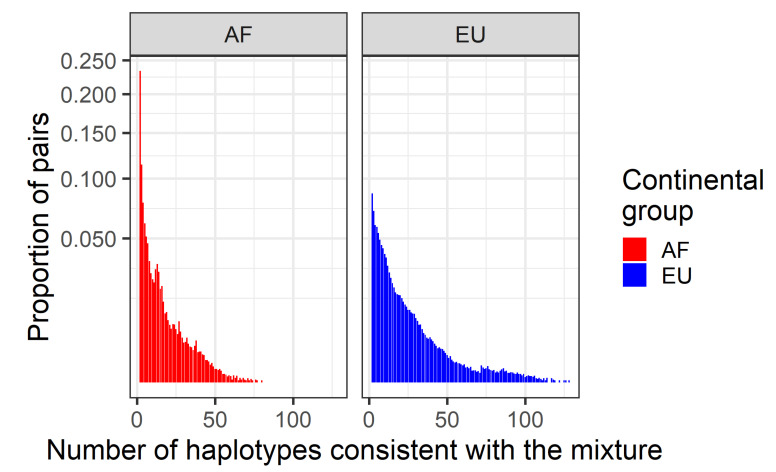
The number of haplotypes consistent with two-person mixtures. Up to 100,000 in silico two-person mitochondrial DNA mixtures from the African (AF, left pane) and European (EU, right pane) continental groups (colors) were created. The number of distinct haplotypes that could not be excluded (*x*-axis) were tabulated and the frequency of such an occurrence (*y*-axis, square root scale) was tabulated. Consistent haplotypes are defined as tracing some path in the variant graph.

HmtDB provides mitochondrial whole genomes as well as their continental group affinities. Individuals associated with European (EU, 11,850 individuals) and African (AF, 3702 individuals) continental groups were taken and two-person in silico mixtures were created. Up to 100,000 mixtures were sought, though owing to the algorithm requirements, slightly less (AF: 92,009, EU: 98,305) were evaluated. Haplotype pairs were excluded if they contained overlapping (but not identical) indel polymorphisms, or if the two individuals sampled happened to have the same haplotype. The haplotypes were sampled from the database with replacement, and then the number of distinct haplotype pairs that can explain the mixture (Figure 9) as well as the number of distinct (i.e., unique) haplotypes that cannot be excluded (Figure 10) was computed.

As shown in Figure 9, the vast majority of two-person mixtures are explained by the single pair of haplotypes used to generate the particular mixture. If mitochondrial DNA haplotypes tend to be unique in a dataset [34], it follows that the same property is true of haplotype pairs. HmtDB data also support a similar trend. For example, in roughly half of the two-person mixtures (AF: 50,620/91,229, EU: 55,561/97,769) both of the haplotypes sampled were unique to the database. In such cases, the classical likelihood of two unknowns from the database may not be well defined (as set *T* is the empty set). Herein lies the advantage of the GBC likelihood as it is both straightforward and well defined in the common case of haplotypes in the mixture that are distinct from the database.

Another interpretation strategy is to consider the proportion of individuals that can be excluded from the mixture. From a graph perspective, a haplotype fails to be excluded if it can be traced in some path in the graph. From a variation perspective, this case generally corresponds to the following: that for all ambiguous sites, the haplotype in question has one of the two allele calls, and in the unambiguous cases, the haplotypes in the mixture and queried, agree. First principles would suggest that most haplotypes have some unique variant that would preclude artificial matches between some query and the mixture. As shown in Figure 10, the mode for both of the AF and EU groups is two, a finding that is consistent with the stated hypothesis. The mean (AF: 9.47, EU: 14.87) and median (AF: 6, EU: 10) number of haplotypes, however, suggest that while the most likely possibility is that some two-person mixture will be associated with only two haplotypes, it is also far more likely that more than two haplotypes will fail to be excluded. This finding is consistent with arguments of [35], which generally favor the more powerful likelihood-based interpretation strategies.

**Figure 11:**
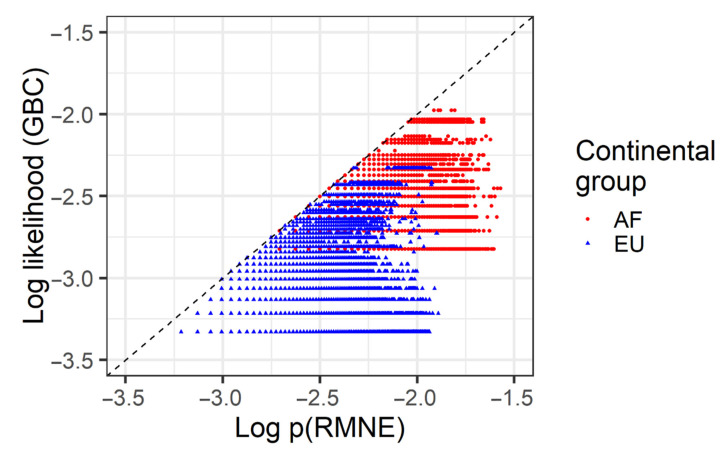
Match statistics of two-person mixtures. The two-person mixtures were created within each of two continental groups (colors, AF: African, EU: European) by sampling haploid sequences from a database. The likelihood of the pair (treating one individual as an unknown, log_10_ transformed, *y*-axis) is contrasted against the probability of the random man not excluded (p(RMNE), log_10_ transformed, *x*-axis).

While Figure 9 and Figure 10 describe the match properties of haplotypes, they fail to describe the rarity of the haplotypes, either individually or in combination. A more formal description of rarity is to assess both the likelihood of a mixture, in this case treating one individual as an unknown, as well as the probability of the random man not excluded (p(RMNE)) (Figure 11). Both of these probabilities depend on the haplotype frequencies as apparent in the database, which for rare alleles is in large part governed by the size of the database (larger in EU than AF, 11,850 EU versus 3702 AF), as well as other population genetic factors such as the population mutation rate (often estimated as nucleotide diversity, wherein AF is larger than EU). For both properties (the database size and the probability of a nonidentical haplotype), large values are associated with smaller likelihoods and smaller p(RMNE)s. (Note that the probability of a nonidentical haplotype is sensitive to the population mutation rate, recent demographics, the variance in female reproductive success and the time to the most recent common ancestor in a given population, amongst other factors: see [31]). The balance in this case seems to favor the size of the database (Figure 11), where the Europeans have both smaller GBC-likelihoods and smaller p(RMNE). This in turn would suggest that while Europeans tend to be more similar to each other than Africans are to each other (~30 versus 55 pairwise differences on average [34]), the individual haplotypes themselves tend to be rare regardless of their population affinities (also see [34] which only found two duplicate haplotypes in these groups and that was in AF). The property of unique haplotypes may hold less well in population isolates and in groups that have experienced recent bottlenecks, which in turn would cause the same haplotype to be shared more widely. Contrasting the likelihood to the RMNE also finds that the likelihood is smaller (the magnitude is reduced by ~0.5) than that of the RMNE, though note that the interpretation of the likelihood is more appropriate when considered as an LR. The likelihood is comparable to the p(RMNE) in some cases, i.e., under the given experiment and at a high level both approaches can be interpreted as an assessment of the rarity of a single contributor (Figure 11). In the case of mitochondrial DNA mixtures, not only are, for example, the expected values more powerful for likelihood-based approaches than for the p(RMNE), but the variance in the p(RMNE) appears to have substantial variance as well.

## 4. Conclusions

This work’s principal focus has been the construction of an algorithm for the analysis of mitochondrial DNA mixtures and a software implementation of such (i.e., MMDIT). However, there are additional considerations for interpreting mitochondrial DNA mixtures and how graph algorithms can support such issues. One such issue is genotyping error, which may occur during the laboratory phase or when interpreting alleles. The RMNE and LR approaches described both permit masking, which allows some regions of the mitochondrial genome to be discarded both in the mixture and in the mitochondrial genomes constructed from the database. Masking occurs simply by removing variation that occurs in a given set of intervals (similar to the approach of SAM2 [21]), so it is best applied in locations that are robust to the ambiguous placement of indel variation. Masking as a proposed solution to genotyping error is only satisfactory if it can be stated a priori that particular regions of the sequence data are too difficult to assess. Undetected or undetectable variants may prove to be a more pernicious issue, especially as the minor allele fraction approaches the detection threshold. Another related complication is the treatment of heteroplasmy variation. The given approach can in principle include heteroplasmies. However, since heteroplasmy varies across tissue types [14,44] and within tissue type (e.g., hairs) [15,45], the presence of heteroplasmy may not be apparent in the single-source sample or in the mixture. Thus, it is entirely possible that heteroplasmies would not match even though the haplotypes themselves originate from the same individual. Future directions to consider may include incorporating uncertainty in genotyping (e.g., by fuzzy string matching) as well as approaches that focus on rare variants that are well-genotyped for mixture interpretation.

## Data Availability

The data used in this study as well as the graph and match statistic codebase are openly available at: https://github.com/Ahhgust/MMDIT.

## Appendix A

EMPOP is an invaluable cutting-edge resource for applications in mitochondrial genomics. The ability to use the mitochondrial gene tree to better inform variant calling is particularly important to the forensic analyst, and with the use of phylogenetic placement and description of sequence variation is instrumental to this endeavor. The properties of mixtures and their description are complicated, as the genotype descriptions of mixtures must extend to an arbitrary number of contributors (much beyond what EMPOP variant descriptions were intended). Other approaches to describing genomic variation may instead be considered, though admittedly the advantages of doing so may be slight. In particular, there are some edge cases to consider when creating variant graphs from variant calls encoded in the manner of EMPOP, and these edge cases become apparent when nearby variants are described. In particular, in the formulation of EMPOP, multibase deletions with respect to the reference may be split into multiple records, which, in turn, creates an ambiguity with mixture interpretation. For instance, a 2bp deletion in one individual can be split into two records, a record for the first deleted base and another record for the second deleted base. If this individual is a contributor of the mixture, however, it is unknown if what is described is two separate deletions (in two separate individuals), or one deletion in each individual. EMPOP also supports multibase or block events, which for mixtures is a critical aspect of the description.

Another example relates to how nearby insertion and deletion polymorphisms are encoded. For example, consider a reference sequence of ACCT and individuals with ACT and ACCCT in a mixture (with this example intending to imitate positions 16,183–16,189 in the rCRS [30]). This example can be encoded as 3c 3.1c (ACCT-> ACT, ACCT -> ACCCT). However, these encodings (deleting a C at position 3, and then inserting a C) are reciprocal, thus a path in the graph would also describe the reference sequence. This is of course problematic unless the rCRS allele (at this location) is truly present in the mixture. Thus, with respect to the graph, care must be taken to ensure that the reference sequence is not encoded in the path unless it is truly present in the mixture. With respect to the format of the variations, the 3c, 3.1c example also contains an ambiguity. If a two-person mixture is assumed (neglecting heteroplasmy), one should reasonably infer that two alleles are present (ACT and ACCCT), to the exclusion of the reference sequence. The ambiguity, however, is that if instead 3 alleles are present (ACT, ACCT, and ACCCT), the exact same sequence differences would be reported (indeed, the differences to the reference sequence are the same, yet the alleles in the mixture are different), which suggests that describing sequence differences alone may at times be insufficient to characterize the alleles present in a suitably complex mixture.

One tractable solution to both the reciprocal operations problem and the sequence difference problem is to describe blocks in the alignment (of arbitrary size), and then to describe the string sequences identified therein. In this manner some range in the rCRS is specified, with the common case referring to single nucleotides, or the space between nucleotides in the reference sequence (insertions). Once the genomic index has been given, then the alleles that are present in the mixture can be described (for the span of this range). This encoding style can be seen in the variant call format (VCF) [46], which has the further advantage of permitting phase and quantification of the allele calls, as well as clearly expressing what alleles are present at each genomic index and which genomic indexes are not callable (and likely with homopolymeric stretches in some chemistries). The VCF file format is not without limitations. For one, from a practical standpoint it is fairly unintuitive, and, for another, it requires the ploidy (in the case of mitochondrial mixtures, this can be taken as the number of haplotypes) to be specified a priori. However, if ploidy is allowed to be loosely interpreted (simply describing what alleles are apparent), and if it is constrained to involve substitution, insertion and deletion polymorphisms over genomic indexes that are non-overlapping (across records), it should be appropriate for the purpose. Additional consideration should also be given to disallow reciprocal operations, which in practice is addressed by considering homopolymeric regions collectively as a single interval (or the equivalent thereof). Relating back to the 3c, 3.1c example, one record could be given in the VCF file (position 3), and the alleles observed could be the empty string and two Cs (if two alleles are deemed present), or all three alleles could be specified if a third reference allele is also observed in the mixture. Such an encoding would also permit a quantitation, which may be useful for describing mixture proportions and the extent of heteroplasmy.
